# SURGICAL OUTCOMES OF VITREOMACULAR TRACTION TREATED WITH FOVEAL-SPARING PEELING OF THE INTERNAL LIMITING MEMBRANE

**DOI:** 10.1097/IAE.0000000000003139

**Published:** 2023-03-13

**Authors:** Francesco Morescalchi, Andrea Russo, Francesco Semeraro

**Affiliations:** Department of Neurological and Vision Sciences, Eye Clinic, University of Brescia, Italy.

**Keywords:** foveal architecture, foveal-sparing peeling, internal limiting membrane, retinal sensitivity, vitreomacular traction

## Abstract

To compare sensitivity of the retina after complete internal limiting membrane (ILM) peeling versus foveal-sparing ILM peeling in vitrectomy for vitreomacular traction syndrome.

**Methods:**

This was a randomized, prospective, comparative study. Thirty-four eyes were randomized to undergo peeling with foveal sparing of the ILM (FS group) or complete peeling group. Foveal and perifoveal retinal sensitivity, visual acuity, and central macular thickness were the main outcome measures.

**Results:**

Parafoveal retinal sensitivity exhibited a significant improvement in both the FS and complete peeling groups (+2.43 ± 0.82 dB and +1.79 ± 0.86 dB, respectively; *P* = 0.037). Significant improvements were observed in both visual acuity and central macular thickness in both groups. No cases of epiretinal membrane recurrence were observed in the FS group.

**Conclusion:**

Both the FS and complete peeling surgical techniques are safe and yielded good anatomical and functional results; however, a significant difference in favor of FS was found in relation to the best-corrected visual acuity and perifoveal retinal sensitivity. Preservation of the foveal ILM disc allowed the anatomical restoration of the foveal architecture in most vitreomacular traction syndrome cases without signs of stiffening or ILM fibrosis over a follow-up period of 1 year.

## Abstract 2

In patients with vitreomacular traction, internal limiting membrane peeling with foveal-sparing leads to better postoperative visual acuity and better perifoveal central retinal sensitivity in comparison with surgery with complete internal limiting membrane peeling.

Aging of the vitreous gel causes a slow liquefaction of its central part and the weakening of the adhesion of the posterior hyaloid from the posterior pole. These two physiological phenomena occur slowly and simultaneously, and lead to posterior vitreous detachment, which occurs commonly between the sixth and seventh decades of life. Vitreomacular adhesion associated with vitreopapillary attachment, in the presence of incomplete posterior vitreous detachment, may cause vitreomacular traction syndrome (VMTs).^1,2^

Visual acuity in VMTs declines by 2 or more Snellen lines in 64% of cases over a median follow-up of 5 years.^3^ Potential complications include cystoid macular edema, epiretinal membranes (ERMs), macula detachment, impending macular hole, and full-thickness macular hole (FTMH).^4–6^ Although spontaneous release of the hyaloid adhesion can occur within 6 to 12 months, case series have shown that VMTs persists in 89% to 47% of patients.^3,7,8^ The intravitreal injection of vitreolytic agents (ocriplasmin) or gas (perfluoropropane or sulfur hexafluoride) may be effective treatments in selected groups of patients.^9,10^

In symptomatic patients, surgical release of the vitreomacular attachment and the resolution of both anteroposterior and tangential traction, by means of pars plana vitrectomy (PPV), has proven to be an effective treatment.^11^ The results of PPV vary according to the morphology and duration of VMTs, with a gain of two or more Snellen visual acuity lines observed in 45% to 100% of eyes treated with PPV.^12^

Internal limiting membrane (ILM) peeling has generally been accepted as a fundamental step for macular surgery, to release all the tangential forces that deform the macula, and also to reduce the recurrence of ERM.^13^ However, in VMTs, the anatomy of the central fovea is often weakened by the formation of internal pseudocystic spaces and by the thinning or disruption of the inner or outer retinal layers. Internal limiting membrane peeling in VMTs could damage the fovea, causing decreased visual function or an iatrogenic macular FTMH. The surgical technique of ILM peeling with foveal sparing has been successfully described in specific types of retinal diseases, including myopic foveoschisis, macular hole, and lamellar macular hole.^14–18^

This prospective study was designed to compare anatomical and functional outcomes of complete ILM peeling (CP) versus foveal sparing (FS) ILM peeling during PPV for VMTs.

## Patients and Methods

### Study Design

This study was completed at the Eye Clinic of “Spedali Civili di Brescia” and complied with the Declaration of Helsinki. The ethics committee approved the study protocol (registered with clinicaltrials.gov, identifier NCT02361645), and all patients signed the informed consent.

The present was a randomized and prospective study involving 34 eyes of 34 participants who were programmed to undergo vitrectomy for VMTs between May 2014 and February 2019. Participants were randomized in a 1:1 fashion to either complete ILM peeling (CP group) or foveal-sparing ILM peeling (FS group).

### Vitreomacular Traction Syndrome Definition

To fulfill with the diagnosis of VMTs, the following aspects need to be present on at least one optical coherence tomography (OCT) slab^18^: (1) partial vitreous detachment, as indicated by the elevation of cortical vitreous above the retinal surface in the perifoveal area; (2) persistent vitreous attachment to the macula within a 3-mm radius from the center of the fovea; (3) an acute angle between the posterior hyaloid and inner retinal surface; (4) the presence of changes in foveal morphology, including distortion of the foveal surface, intraretinal structural changes such as pseudocyst formation, elevation of the fovea from the retinal pigment epithelium, or a combination of any of these three features; (5) the absence of full-thickness interruption of all retinal layers. This study included both focal VMT (width of attachment ≤1,500 *µ*m) and broad VMT (width of attachment >1,500 *µ*m).

The anatomical configuration of VMTs was classified according Lee et al^19^ into 4 types: (1) VMTs with foveal pseudocyst, (2) VMTs with parafoveal retinoschisis, (3) VMTs with outer retinal dehiscence of the fovea, and (4) other types.

### Inclusion and Exclusion Criteria

Eligible patients met with the following: (1) the provision of written informed consent, (2) the presence of VMTs recognized by optical coherence tomography (OCT; OPKO/OTI, Miami, FL, USA), and (3) visual disturbances persisting for at least six months associated with a best-corrected visual acuity (BCVA) of less than logMAR 0.20 (20/32). Patients were excluded for: (1) any prior vitreoretinal operation, including intraocular injections, (2) high myopia (>7 diopters), (3) age-related macular degeneration, (4) glaucoma diagnosis, and (5) diabetic retinopathy or any other retinopathy. Noticeably, patients with advanced cataract (higher than NO2, C2, P1 as per the LOCS III cataract classification) were likewise rejected in order not to interference the microperimetry evaluation.

### Clinical Evaluation

Comprehensive ocular and medical anamnesis were acquired along with a comprehensive eye examination. At each appointment, a blinded examiner performed the following assessments: (1) BCVA using a ETDRS chart, (2) dilated fundus biomicroscopy, (3) OCT examination of central retinal thickness, and (4) combined OCT topography and microperimetry (OPKO/OTI). This method allowed the assessment of central retinal sensitivity wherein, as previously described.^20,21^

### Surgical Technique

All phakic patients underwent uneventful clear lens phacoemulsification with IOL implantation in-the-bag immediately before vitrectomy, to prevent the lens opacification that often occurs in elderly patients several months after a complete vitrectomy.

After cataract surgery, a 25-gauge, three-port PPV was completed as described below.

First, careful induction of a Weiss ring over the optic disc took place. Then, the posterior hyaloid was removed with the vitrectomy probe circumscribing the vitreous and cutting it in a circular fashion around the fovea, to prevent tearing of the fovea.

**Fig. 1.**
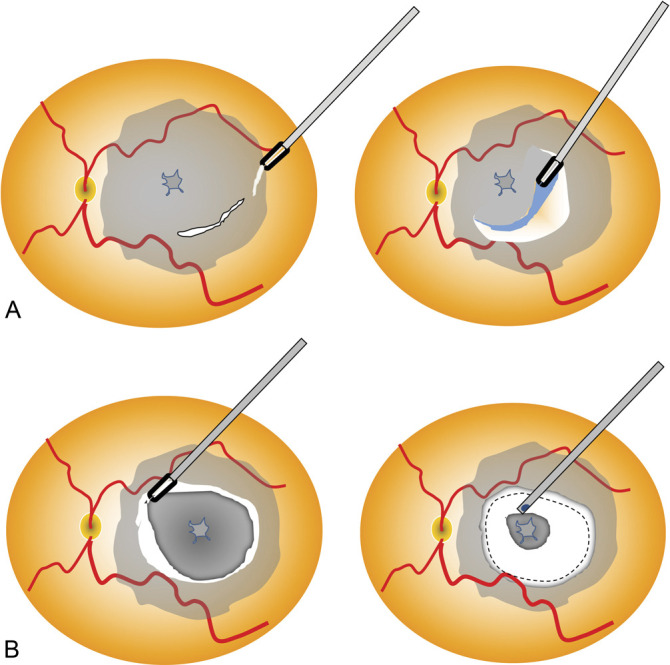
(**A**) Complete peeling of the ILM. In the foveal-sparing procedure (**B**), the ILM is grasped several times near the arcades and pulled in a centripetal fashion. The remaining floating flap is finally trimmed with the vitrectome.

To visualize the ILM (and possibly the ERM), initial staining with Membrane Blue-DUAL (DORC, Zuidland, The Netherlands) was achieved. In the CP group, the ILM was entirely peeled up to the arcades; whereas in the FS group, the peeling of the ILM was initiated next to the arcades and extended in a centripetally toward the center of the fovea, until the beginning of the orange pigmentation of the fovea. A disc of ILM with diameter of about one optic disc was left in place over the fovea (Figure 1A), and the vitrectome was used to cut its floating edges, lowering the aspiration and the cut rates (to 150 mmHg and to 1,500 cuts/minute, respectively; Figure 1B). The vitrectomy was than completed with a careful shaving of the vitreous base to obtain a complete and safe air tamponade.

The retina was then inspected with scleral depression, and any iatrogenic retinal holes or tears were treated with argon laser photocoagulation. At the end of the surgery, in both groups, the vitreous chamber was filled with air. The patients were requested to remain in a face-down position for at least two days. All surgeries were performed by the same retinal vitreous surgeon (F.M.).

### Statistical Analysis

A repeated measures ANOVA with Bonferroni and Greenhouse–Geisser correction was used to detect statistically significant changes in BCVA, central retinal thickness, and perifoveal retinal sensitivity (pFRS) after the surgery. Independent-samples *t*-test was applied to determine if the intergroup means were significantly different. The SPSS v22.0 software statistical package (SPSS, Chicago, IL, USA) for Windows was used.

## Results

### Baseline Characteristics

Sixteen patients in the FS group and all 17 patients in the CP group completed the follow-up period of 12 months and were included in the statistical analysis.

The baseline characteristics of the CP and FS groups are shown in Table 1. No significant differences were found between the two groups in terms of BCVA, age, sex, lens status, central macular thickness, pFRS, VMTs extension (focal/broad), and type (presence of foveal pseudocyst, parafoveal retinoschisis, and dehiscence of the fovea).

**Table 1.** Patient Demographics and Baseline Characteristics

	Complete Peeling (N = 17)	Foveal Sparing (N = 16)	*P*
Age: mean (±SD), years	67.7 (8.0)	70.1 (4)	0.1
Sex: n (%)			
Men	5 (29.4)	6 (37.5)	—
Women	12 (70.6)	10 (62.5)	—
BCVA: mean (±SD)			
LogMAR	0.54 (0.1)	0.56 (0.2)	0.7
Snellen	20/69	20/72	
CMT (±SD), µm	426 (129)	430 (130)	0.8
pFRS (±SD), dB	11,0 (2.4)	11.5 (2.1)	0.4
VMT extension			
Focal	8	7	—
Broad	9	9	—
Lens status: n (%)			
Pseudophakic	2 (11.8)	3 (18.8)	—
Phakic	15 (88.2)	13 (81.3)	—
VMT type (%)			
Foveal pseudocyst	8 (47.1)	7 (43.8)	—
Parafoveal retinoschisis	4 (23.5)	4 (25.0)	—
Dehiscence of the fovea	3 (17.6)	3 (18.8)	—
Others	2 (11.8)	2 (12.5)	—

BCVA, best-corrected visual acuity; CMT, central macular thickness; logMAR, logarithm of the minimum angle of resolution; pFRS, perifoveal retinal sensitivity.

### Postoperative Functional Outcomes

The mean postoperative BCVA in the CP group improved significantly from logMAR 0.54 (20/69) before surgery to logMAR 0.27 (20/37) after surgery (*P* = 0.02). Similarly, the mean postoperative BCVA in the FS group improved significantly from logMAR 0.56 (20/72) before surgery to logMAR 0.14 (20/27) after surgery (*P* < 0.001). The mean improvement was similar between the two groups (*P* = 0.68). Table 2 shows the postoperative outcomes of the CP and FS groups.

**Table 2.** Changes in Visual Acuity, Central Macular Thickness, and Parafoveal Retinal Sensitivity Over 12 Months After Surgery

	Complete Peeling (N = 17)	Foveal Sparing (N = 16)	*P*
Overall BCVA: mean (±SD)			
LogMAR	0.27 (0.17)*	0.14 (0.15)*	0.027
Snellen	20/37	20/27	
Mean logMAR improvement	0.27 (0.07)	0.42 (0.05)	<0.001
VMT extension: mean logMAR (±SD)			
Focal	0.29 (0.11)*	0.20 (0.20)*	0.25
Broad	0.24 (0.19)*	0.10 (0.07)*	0.32
VMT subtype: mean logMAR (±SD)			
Foveal pseudocyst	0.31 (0.18)*	0.22 (0.17)*	0.64
Parafoveal retinoschisis	0.17 (0.09)*	0.07 (0.06)*	0.23
Foveal dehiscence	0.18 (0.12)*	0.07 (0.11)*	0.45
Others	0.22 (0.08)*	0.08 (0.11)*	0.21
Lens status: mean logMAR (±SD)			
Phakic	0.29 (0.18)*	0.16 (0.15)*	0.04
Pseudophakic	0.18 (0.04)*	0.02 (0.03)*	0.04
CMT (±SD), *µ*m	225 (61)*	241 (45)*	0.43
Mean CFT reduction (±SD), *µ*m	201 (67)	189 (85)	0.68
pFRS (±SD), dB	12.79 (1.8)*	13.93 (1.4)*	0.05
Mean pFRS increase (±SD), dB	+1.79 (1)	+2.43 (0.63)	0.03

* Statistically significant differences compared with the baseline.

BCVA, best-corrected visual acuity; CFT, central foveal thickness; CMT, central macular thickness; logMAR, logarithm of the minimum angle of resolution; pFRS, perifoveal retinal sensitivity.

**Fig. 2.**
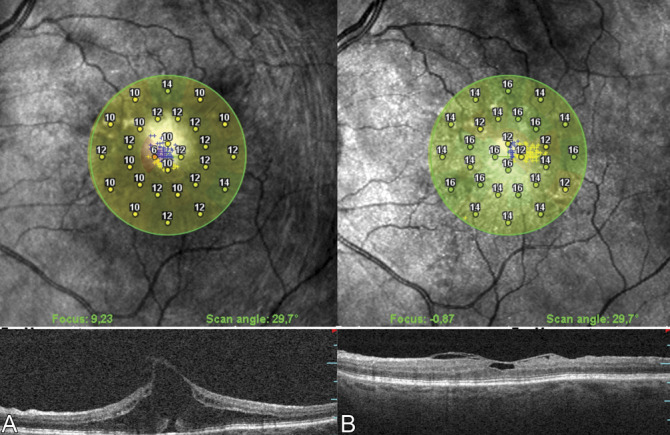
Representative microperimetry image of a patient with VMT syndrome before (**A**) and 12 months after vitrectomy with peeling with foveal sparing of the ILM (**B**). The preoperative perifoveal retinal sensitivity (pFRS) was 9.5 dB, which increased to 14 dB at 12 months after surgery.

Best-corrected visual acuity improved in both broad and focal VMTs, without distinction between the various VMTs subtypes, either using the FS or CP technique. However, overall the final BCVA data showed a significant difference between CP and FS groups (*P* = 0.027), with the FS group showing a better improvement after the surgery. In both groups, a significant reduction of the central retinal thickness and a significant improvement in the mean microperimetry pFRS was observed (Figure 2).

The difference in pFRS between the two groups was at the limit of significance (*P* = 0.05), whereas the mean increase in pFRS in the central 4° was greater in the FS group (+2.43 ± 0.82 dB vs. +1.79 ± 0.86 dB; *P* = 0.037).

**Fig. 3.**
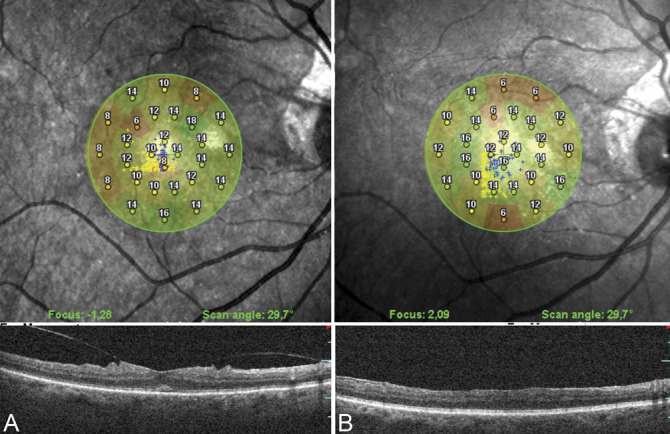
Representative microperimetry image of a patient with VMT syndrome before (**A**) and 12 months after vitrectomy with peeling with foveal sparing of the ILM (**B**). The preoperative perifoveal retinal sensitivity (pFRS) was 11 dB, which increased to 13.5 dB 12 months after surgery. Note the appearance of some areas with relative scotomata not present in the preoperative examination.

In three patients in the CP group (17.6%) and six patients in the FS group (37.5%), some asymptomatic paracentral relative scotomata (sensitivity ≤6 dB), which were not present before surgery, were found (Figure 3). Given the limited number of points testes by the microperimetry pattern, we were unable to correlate these microscotomas with the anatomical alterations of the macula.

### Postoperative Anatomical Findings

**Fig. 4.**
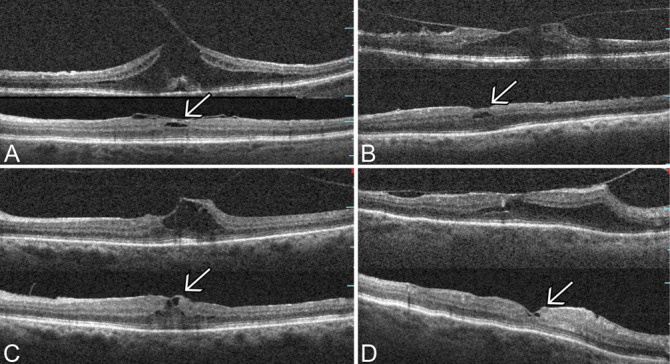
Representative OCT scans for four eyes that underwent vitrectomy with foveal sparing peeling of the ILM for VMT. In each quadrant, the upper image shows the preoperative appearances, and the lower image shows the appearance 12 months after surgery. The arrow indicates the residual ILM above the fovea. (**A**) A 70-year-old woman, preoperative BCVA: 0.52 logMAR (20/66); postoperative BCVA: 0.15 logMAR (20/28). (**B**) A 61-year-old woman, preoperative BCVA: 0.39 logMAR (20/49); postoperative BCVA: 0.07 logMAR (20/23). (**C**) A 69-year-old woman, preoperative BCVA: 0.63 logMAR (20/85); postoperative BCVA: 0.22 logMAR (20/33). (**D**) A 71-year-old woman, preoperative BCVA: 0.45 logMAR (20/56); postoperative BCVA: 0.09 logMAR (20/24).

At the end of the follow-up period, the morphology of the macula improved in a variable way according to the deformation present before the intervention. In the FS group, the OCT scans showed ILM residues lying above the fovea, reformation of the foveal depression in 12 eyes (75%), and constant reduction of the foveal intraretinal cystic spaces throughout the follow-up period. Notably, the thickness of the fovea remained unchanged over time (Figure 4).

**Fig. 5.**
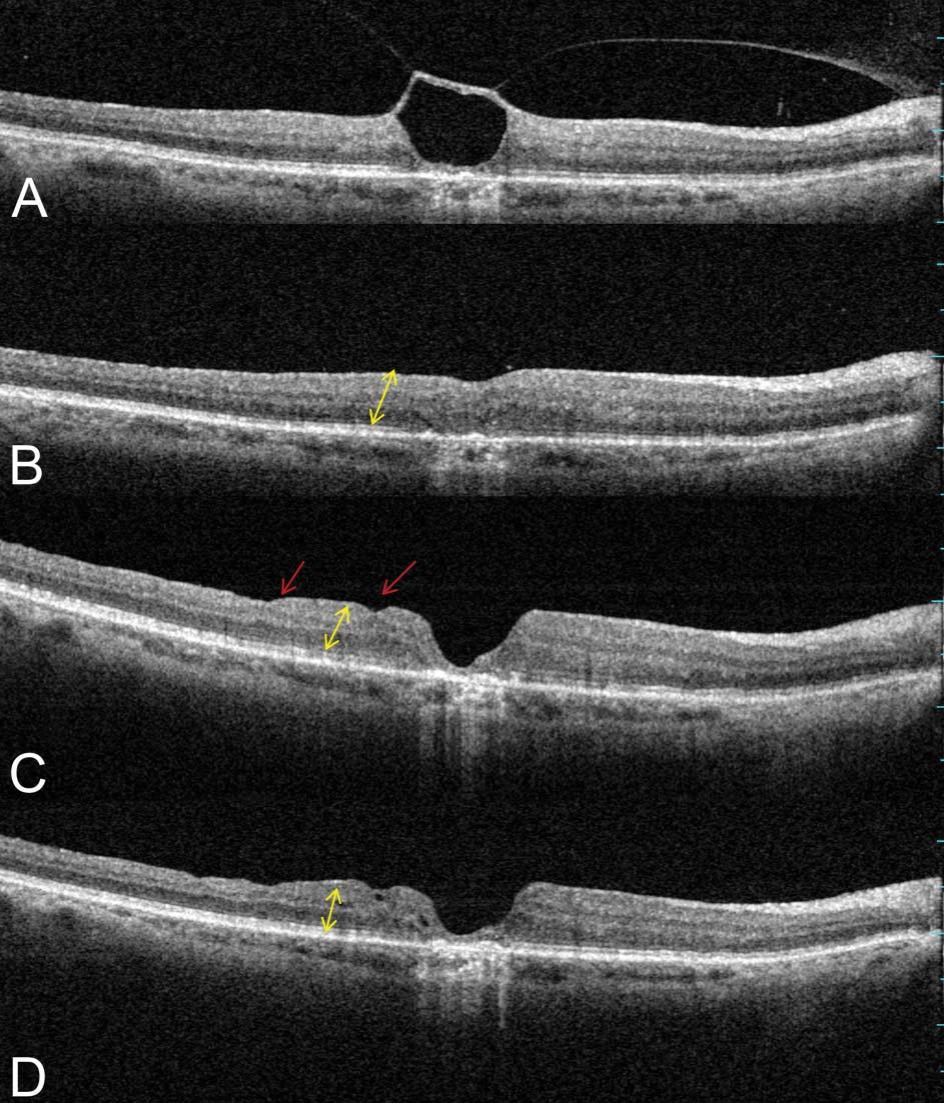
Representative OCT scans of a 69-year-old woman that underwent vitrectomy with CP of the ILM for VMT. (**A**) Preoperative appearances, BCVA: 0.82 logMAR (20/132); (**B**) two months after surgery, BCVA: 0.52 logMAR (20/66); (**C**) six months after surgery, BCVA: 0.52 logMAR (20/66). Nicks and dimples in the inner retinal layers can be seen extending over the center of the macula (red arrows); (**D**) 12 months after surgery, BCVA: 0.52 logMAR (20/66). Interestingly, the fovea shows progressive thinning over time (yellow arrows).

In the CP group, the foveal profile improved gradually, with the reformation of the foveal depression in 15 eyes (88%). However, thinning of the foveal retinal layers was evident in five patients after 12 months of follow-up (Figure 5).

Central macular thickness significantly improved in both groups, from a preoperative mean of 426 ± 129 *µ*m for CP and 430 ± 130 *µ*m for FS to a postoperative mean of 225 ± 61 *µ*m for CP and 241 ± 45 *µ*m for FS (*P* < 0.01). No significant difference was found between the CP and FS groups in terms of preoperative (*P* = 0.8) and postoperative (*P* = 0.4) central foveal thickness.

### Intraoperative and Postoperative Complications

**Fig. 6.**
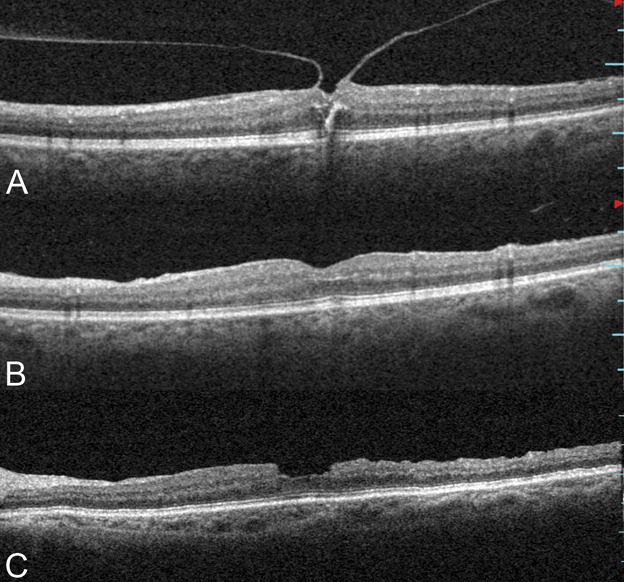
Optical coherence tomography scans of a 67-year-old woman who underwent complete ILM peeling. During the ILM peeling, an iatrogenic FTMH formed intraoperatively. The ILM peeling procedure was enlarged, and the eye was filled with 20% sulfur hexafluoride. The patient was positioned face down for a week. Preoperatively (**A**), VMT syndrome was visible; 1-month postoperatively (**B**), the FTMH closed, and the BCVA improved from 0.39 logMAR (20/49) to 0.22 logMAR (20/33); and 1-year postoperatively (**C**), an evident reduction in the thickness of the central retinal layers was appreciable.

In one patient enrolled in the FS group, the ILM was obstinately attached to the posterior hyaloid and detached from the fovea during the peeling; he was therefore excluded from the study. In one patient from the CP group, during the ILM peeling, an iatrogenic FTMH was created intraoperatively. The ILM peeling procedure was therefore enlarged, and the eye was filled with 20% sulfur hexafluoride (SF6) with face down positioning for a week. The macular hole closed after the surgery, and the BCVA improved from 0.39 logMAR (20/49) to 0.22 logMAR (20/33). After 1 year, the OCT examination showed an evident reduction in the thickness of the central retinal layers (Figure 6).

**Fig. 7.**
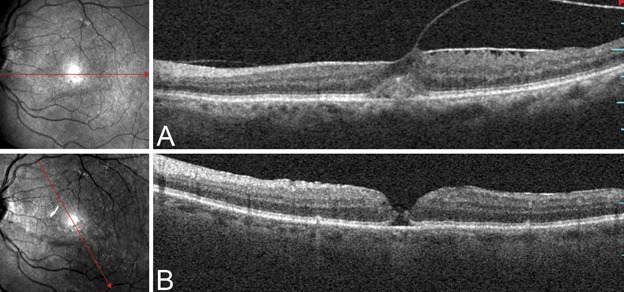
Optical coherence tomography scan of a 65-year-old male patient with a broad VMT with dehiscence of the fovea, which was treated with phacoemulsification of the lens and PPV with complete ILM peeling. (**A**) Preoperative OCT scan showing a partial posterior vitreous detachment temporal to the fovea and a posterior hyaloid attached nasally. (**B**) The OCT scan performed during the follow-up visit a week after the surgery showing a secondary FTMH. A second PPV was performed one month after the first operation. The area of ILM peeling was enlarged, and the eye was filled with 20% sulfur hexafluoride. The patient maintained a face-down position for seven days. The closure of the macular hole occurred after the first week after the second PPV. Preoperative BCVA was 0.55 logMAR (20/70); postoperatively, it was 0.39 logMAR (20/49) at the end of the follow-up period.

A postoperative FTMH formed in the eye of another patient belonging to the CP group. It was diagnosed with the OCT examination performed during the follow-up visit a week after the surgery. This patient had broad VMTs with dehiscence of the fovea, and a partial posterior vitreous detachment temporal to the fovea that was still attached nasally (Figure 7).

A second PPV had to be performed one month after the first operation in which the area of ILM peeling was enlarged, and the eye was filled with 20% SF6. The patient maintained a face-down position for seven days. The closure of the macular hole occurred after the first week of the second PPV, and the BCVA showed a moderate improvement at the end of the follow-up period (from 0.55 to 0.39 logMAR).

None of the patients in the FS group suffered from ERM recurrence in the foveal area at the end of the 12-month follow-up period. No patient had any retinal complications (retinal breaks or retinal detachment) during follow-up.

## Discussion

This study showed that both surgical techniques are safe and can yield good anatomical and functional results; however, a significant difference favoring FS was found in relation to BCVA and pFRS. Indeed, saving the foveal ILM during ILM peeling in the VMTs allows better results to be obtained regarding the improvement in the BCVA, compared with complete ILM peeling.

Studies conducted on the physiopathology of VMTs have shown that a “double layer” of preretinal proliferation (epiretinal tissue and vitreous collagen) are often present above the ILM in patients with VMTs.^22^ In VMTs, the vitreous may be tightly anchored to the fovea by the extension of a “mild” ERM onto the posterior hyaloid.^23^ This ERM bonding precludes atraumatic vitreo foveal separation by the surgical plane made from the hyaloid and the retina. The vitreous can be separated from the fovea only on peeling the associated ERM and ILM.^23^ Moreover, the presence of ERMs, by stiffening the ILM, increase the occurrence of structural changes of the neuroretina, causing negative effects on quality and quantity of vision.^22^ In these studies, it is postulated that ILM removal can completely relieve the tangential tractional forces on the macular area and minimize the recurrence of ERM.^13^ Therefore, the simultaneous removal of ERM, followed by ILM peeling, has become the standard treatment for VMTs.^24,25^

However, the complete removal of the ILM could also damage the fovea because the Müller cells are closely connected to the ILM and could be damaged by this surgical procedure.^26^

This study revealed that there were functional differences depending on whether or not the ILM is surgically detached from the macula in cases of VMTs. We recorded differences in the BCVA and in the microperimetry results between the two groups, obtaining a greater mean BCVA improvement and a greater pFRS in the FS than the CP. This finding is consistent with other studies where FS was performed for other traction maculopathies. The FS procedure has been evaluated for its use in the management of FTMH^16–18^ and has also given good results in the treatment of myopic traction maculopathy,^17^ lamellar macular hole,^27^ and idiopathic ERM.^20^

Müller cells are the main glial cells of the retina and have an active role in retinal function. They possess a cell body allocated in the inner nuclear layer and a multitude of cellular processes that span the entire thickness of the neurosensory retina. They form the inner retinal surface at the level of the ILM and contact and wraps every type of neuronal cell body and process to the photoreceptor layer, where they enlarge and form the external limiting membrane. The inner segment of the central fovea is composed of an inverted “cone-shaped” zone, referred to as the Müller cell cone, where the ILM and external limiting membrane are strictly connected. The Müller cell cone can be avulsed by VMT in the pathogenesis of idiopathic FTMH. These morphological relationships reflect a multitude of functional interactions between Müller cells and neurons such as extracellular ion homeostasis, glutamate recycling, and the exchange of waste products with the underlying ganglion cells. It has also been suggested that Müller cells could also aid light transmission to photoreceptors by acting as optical fibers.^28^

Virtually any surgical damage to Müller cells can alter retinal function; if damage occurs to the Müller cells over the fovea, the effect on vision could be exponentially greater. Hence, there is a theoretical advantage to save the ILM over the fovea as much as possible, and this explains the increased visual function in patients in whom the ILM on the fovea was spared.

A possible postoperative complication of PPV for VMTs is the onset of an iatrogenic FTMH secondary to the surgical procedure. In the CP, we recorded the onset of a FTMH intraoperatively, during the ILM peeling, and another case of secondary FTMH that arose a week after the surgery (incidence of FTMH: 11% in the CP). No cases of intraoperative or postoperative FTMH occurred in the FS.

Surgery for VMTs is riskier than surgery for ERM, because in the latter the fovea is thickened, and there is no risk of unroofing the fovea. On the contrary, the fovea in the patient with VMTs is often thinned, weakened, and edematous. Traction during ILM peeling, together with the loss of the substance constituted by the ILM itself, risks the unroofing of the center of the foveola and the formation of a FTMH.

The Authors believe that it is essential to remove the ILM in VMTs because the ILM is often damaged or broken in the center. In the postoperative phase, the processes of reactive gliosis over the broken ILM could create traction in a centrifugal direction that could favor the formation of an ERM or a FTMH in turn.^29^

The ILM over the fovea is anatomically very thin and is less accountable for the rigidity of the macula compared with the thicker perifoveal ILM. The surgical removal of a ring of peripheral ILM may interrupt the continuity between peripheral and central ILM, leaving little or no rigidity over it.

The main risk associated with FS is the stimulation of a reactive gliosis of Müller cells that could cause the recurrence of an ERM several months after the surgery. In ERM surgery using the FS technique, ERM recurrence was evident in more than 25% of the cases.^20^

In VMTs, such as in idiopathic FTMH treated with FS technique, the appearance of the fovea returned approximately normal in a significant percentage of cases, and we did not record any recurrence of a secondary ERM during the 12 months of follow-up.

This is probably because of the difference in the pathophysiology of idiopathic ERM compared with that of VMTs and FTMH. In the latter the anteroposterior traction is predominant, whereas the tangential traction because of the epiretinal tissue is less relevant. However, further follow-up is required to confirm the actual absence of this complication.

In conclusion, in this prospective pilot study of 33 consecutive patients with VMTs, we performed ILM peeling while sparing the central ILM over the fovea in 16 eyes, and observed better postoperative BCVA and better postoperative perifoveal central retinal sensitivity in comparison with 17 patients in whom complete ILM peeling was performed.

Our study has a few important limitations: first, the number of enrolled patients was limited. Second, the presence of possible confounding variables such as the state of the lens and the simultaneous presence of different types (focal and broad) and subtypes (foveal pseudocyst, parafoveal retinoschisis, dehiscence of the fovea, etc.) of VMTs. Third, the incomplete evaluation of the preoperative and postoperative macular function because we did not track changes in visual quality.

To the best of our knowledge, it is the first time that this technique has been performed in VMTs. Preservation of the foveal ILM disc allowed anatomical restoration of the foveal architecture in most of the cases of VMTs, without signs of stiffening or ILM fibrosis, over a follow-up period of 1 year. The encouraging outcomes of this study led our group to forsake the CP procedure in favor of the FS procedure for the surgical treatment of routine cases of VMTs. FS procedure, although needing a steeper learning curve than conventional CP, might be effectively performed for those eyes with VMT syndrome particularly prone to mechanical and metabolic damages from comorbidities such as pathologic myopia, glaucoma, and diabetes.
